# Clinical Measures of Bulbar Dysfunction in ALS

**DOI:** 10.3389/fneur.2019.00106

**Published:** 2019-02-19

**Authors:** Yana Yunusova, Emily K. Plowman, Jordan R. Green, Carolina Barnett, Peter Bede

**Affiliations:** ^1^Department of Speech Language Pathology, University of Toronto, Toronto, ON, Canada; ^2^Hurvitz Brain Sciences Research Program, Sunnybrook Research Institute, Toronto, ON, Canada; ^3^Volcal Tract Visualization Lab, Toronto Rehabilitation Institute, University Health Network, Toronto, ON, Canada; ^4^Swallowing Systems Core, Department of Speech, Language, and Hearing Sciences, University of Florida, Gainesville, FL, United States; ^5^Department of Communication Sciences and Disorders, MGH Institute of Health Professions, Boston, MA, United States; ^6^Speech and Hearing Biosciences and Technology Program, Harvard University, Cambridge, MA, United States; ^7^Division of Neurology, Department of Medicine, University of Toronto and University Health Network, Toronto, ON, Canada; ^8^Institute of Health Policy, Management and Evaluation, Dalla Lana School of Public Health, University of Toronto, Toronto, ON, Canada; ^9^Computational Neuroimaging Group, Academic Unit of Neurology, Trinity College Dublin, Dublin, Ireland

**Keywords:** amyotrophic lateral sclerosis, Bulbar ALS, outcome assessment (Health Care), dysphagia, dysarthria, COSMIN

## Abstract

Bulbar impairment represents a hallmark feature of Amyotrophic Lateral Sclerosis (ALS) that significantly impacts survival and quality of life. Speech and swallowing dysfunction are key contributors to the clinical heterogeneity of ALS and require well-timed and carefully coordinated interventions. The accurate clinical, radiological and electrophysiological assessment of bulbar dysfunction in ALS is one of the most multidisciplinary aspects of ALS care, requiring expert input from speech-language pathologists (SLPs), neurologists, otolaryngologists, augmentative alternative communication (AAC) specialists, dieticians, and electrophysiologists—each with their own evaluation strategies and assessment tools. The need to systematically evaluate the comparative advantages and drawbacks of various bulbar assessment instruments and to develop integrated assessment protocols is increasingly recognized. In this review, we provide a comprehensive appraisal of the most commonly utilized clinical tools for assessing and monitoring bulbar dysfunction in ALS based on the COnsensus-based Standards for the selection of health Measurement INstruments (COSMIN) evaluation framework. Despite a plethora of assessment tools, considerable geographical differences exist in bulbar assessment practices and individual instruments exhibit considerable limitations. The gaps identified in the literature offer unique opportunities for the optimization of existing and development of new tools both for clinical and research applications. The multicenter validation and standardization of these instruments will be essential for guideline development and best practice recommendations.

## Introduction

ALS is a relentlessly progressive neurodegenerative disease with considerable clinical heterogeneity compared to other neurodegenerative conditions. Bulbar impairment (oro-motor, dysarthria and dysphagia) is a hallmark feature of the disease and has been associated with the condition since its earliest descriptions (1). While only approximately 30% of patients exhibit bulbar symptoms at onset, the majority of patients develop speech and swallowing difficulties with disease progression. Bulbar signs and symptoms play an important role in the diagnosis of ALS and pose unique management challenges. Bulbar presentation has been associated with shorter survival (2, 3), faster functional decline (4), reduced quality of life (5–7) and increased multidisciplinary support needs (8, 9). Dysarthria has been consistently associated with low mood, withdrawal from activities and social isolation (10, 11). Dysphagia in ALS may lead to weight loss, malnutrition, dehydration, aspiration pneumonia, hospitalization and reduced quality of life (12, 13). Despite these important sequelae, bulbar impairment in ALS is relatively understudied, and the research literature is sparse (14). Proxies of bulbar impairment are underrepresented among outcome measures in clinical trials (15). Validated diagnostic, monitoring and prognostic markers of bulbar dysfunction are lacking and clinical assessment practices vary considerably across various centers (16).

Assessment measures are broadly classified as “diagnostic” when their primary purpose is to confirm the diagnosis, exclude mimics, or classify individual patient according to disease-onset. Some measures have been optimized to characterize symptom severity, while other indices are primarily used to monitor longitudinal change. Depending on the primary purpose of a measure, it is subject to a specific set of requirements defined by the COnsensus-based Standards for the selection of health Measurement INstruments (COSMIN) guidelines (17). These require that, in order to reliably integrate assessment tools into clinical practice, their measurement properties need to be firmly established relative to their primary purpose. All tests need to be assessed for validity and reliability (reproducibility). Diagnostic and screening tests should also be evaluated for their detection abilities (i.e., sensitivity/specificity). Discriminative measures need to be able to detect group differences and measures proposed to track longitudinal change need to be assessed for their ability to capture progressive changes.

The objective of this paper is to provide a review of established bulbar measures in ALS from a diagnostic, screening and disease monitoring perspective. This work is not intended as an exhaustive review of all available measures of bulbar impairment in ALS but as a summary of the current state of the field and its most pressing needs.

## Tools for Diagnosing and Screening for Bulbar ALS

Table 1 provides a summary of tools primarily used for the diagnosis of bulbar dysfunction in ALS highlighting their main advantages and limitations.

**Table 1:** Tools for diagnosis of bulbar signs or ALS.

**Tool**	**Description**	**Advantages**	**Limitations**	**Recommendations**
1. Cranial Nerve Examination	Bulbar motor UMN and LMN signs: weakness, fasciculations, atrophy, tone, reflexes	Well established; (18) easy to perform; reliability of items established in stroke, (19) structural disorders (20) and mixed neurological populations; (21) validated with respect to detection of dysphagia with VFSE	Not sufficiently standardized, subjective; measurement properties have not been evaluated in ALS	Individual items and the test as a whole require further standardization and better testing of measurement properties
2. Needle EMG, Genioglossus, Sternocleidomastoid (SCM), Trapezius	Indicates acute and chronic denervation in selected muscles, LMN changes	Well established, helpful in the exclusion of mimics able to detect subclinical involvement; can be quantitative	Invasive, not well standardized across clinics, requires substantial training, low sensitivity due to difficulty with relaxation (tongue < SCM < trapezius); (22) commonly qualitative	Requires further standardization and establishment of quantitative measures
3. Clinical MRI of bulbar regions (e.g., brainstem, bulbar region of the PMC)	The clinical role of MRI is to rule out neurological mimics, tongue and pharyngeal pathology	Widely available, noninvasive, potentially sensitive to prodromal stages of bulbar disease	Qualitative, not formally assessed with respect to clinical utility in the diagnosis of early stages of bulbar disease	Requires further research effort in establishing clinical utility
4. Auditory perceptual assessment of dysarthria types	Detection of UMN vs. LMN signs in speech/ voice tasks; rating specified dimensions of voice/ speech quality on a Likert scale	Well-established method in dysarthria assessment in SLP	Specific set of ALS-relevant items is not established; (23, 24) may not be equally sensitive to all dysarthria severities; requires specialized training; not well utilized in neurology; lengthy; low reliability for some items (25)	Items require further identification and standardization in ALS as well as better testing of measurement properties
5. Frenchay Dysarthria Assessment	Comprehensive assessment of bulbar structure and function (goals and item overlap with #1 and #4)	Well-established method in dysarthria assessment in SLP; reliability is established (26)	Relatively lengthy; not specific to ALS; validation is limited	Items require further identification and standardization as well as better testing of measurement properties
6. Videofluoroscopic Swallowing Exam (VFSE)	“Gold standard” dysphagia assessment to directly visualize swallow safety and efficiency	Well established in ALS; showed sensitivity to prodromal stage of dysphagia and sensitivity to change (27–29)	Requires expensive instrumentation and highly trained personnel; involves radiation exposure (minimal); need for a separate and additional test	Recommended clinically in patients demonstrated high risk of dysphagia to diagnose and test impact of strategies for treatment planning
7. EAT-10	Screening tool for dysphagia; 10 items; self-administered, symptom-based	Validated and reliability assessed in a large non-ALS cohort; differentiated safe vs. unsafe swallowers in ALS; cut-off of 8 or higher indicates high likelihood of dysphagia (3.1 times); sensitivity 86%, specificity 76%; (30) quick and easy, low administrative burden for scoring	Subjective; may not be sensitive to early disease stages	Recommended for use in clinic as a screener for the presence of dysphagia in ALS.
8.3oz Swallow Test	Screening tool for dysphagia; 3oz of water given to patient in a cup; Pass / Fail; fail includes inability to drink without stopping, cough or throat clear, “wet” voice	Validated in general patient populations with very high sensitivity but poor specificity; quick and easy to use	Not validated in ALS; may miss patients with sensory deficits (silent aspirators) and early signs of dysphagia, (31) could also overestimate dysphagia risk due to “maximum performance” nature of test	Requires further standardization as well as better testing of measurement properties
9. Voluntary Cough	Screening tool for airway defense physiologic capacity using airflow spirometry or peak cough flow meter	Objective, instrumental; validity, reliability, sensitivity (up to 90%) and specificity (up to 82%), depending on the measure, relative to VFS, have been established in ALS (32)	Requires instrumentation and a trained examiner; voluntary cough is mediated differently neurologically to a reflexive cough; effort dependent	Recommended for use in clinic as a screener to index airway defense capacity to expel tracheal aspirate or secretions in ALS

### Cranial Nerve Exam (CNE)

Clinical evidence of upper motor neuron (UMN) and lower motor neuron (LMN) degeneration is required for the diagnosis of ALS. With regards to bulbar impairment, clinical UMN signs include pathological reflexes (e.g., brisk jaw jerk, gag, and other facial reflexes) (18) and LMN signs encompass muscle weakness, atrophy and fasciculations in the jaw, face, tongue and palate (33). Although the clinical neurological examination remains “the best way to localize neurodegeneration *in vivo* and to follow the process in real time,” (34) and the reliability of CNE has been evaluated in various neurologic populations (21, 35), the quantitative psychometric profile of CNE i.e., inter and intra-later reliability, sensitivity, specificity, and responsiveness, have not been systematically evaluated in ALS to date. This represents a research priority for the standardization of assessments.

### Needle EMG

The role of electromyography (EMG) in ALS is the confirmation of acute and chronic denervation. The former may be evidenced by fibrillations, positive sharp waves and fasciculation potentials, which in the tongue are not readily detectable since complete relaxation is difficult to achieve (22). Polyphasic motor unit potentials (MUPs) with prolonged duration, increased amplitude and decreased recruitment are suggestive of chronic denervation. Quantitative motor unit action potential analysis in subclinical bulbar involvement is thought to be superior to peak ratio interference pattern analysis (36). Depending on local protocols, the genioglossus is the most commonly assessed muscle (37), but the evaluation of the sternocleidomastoid (38), masseter, temporalis, frontalis (39), mentalis (40), and trapezius (22) muscles have also been proposed to resolve diagnostic uncertainty. While Motor Unit Number Estimation (MUNE) techniques (41, 42), such as MUNIX (43) have been extensively utilized to quantify motor neuron loss in the limbs, they have only been relatively recently adopted to assess the denervation of the tongue (44) and further development is required for their acceptance to clinical practice.

### Clinical Neuroimaging

While brain imaging is not required to establish the diagnosis of ALS, MRI is commonly used as part of the diagnostic work-up to rule out alternative neurological conditions which may mimic ALS (45, 46). In bulbar onset patients the careful evaluation of the brain stem for structural, neoplastic, vascular, inflammatory and infiltrative processes is particularly important. Pathological processes superior to the brainstem; demyelination, neurovascular syndromes, neurosarcoidosis, leukodystrophies, malignancies, and neurodegenerative conditions may also manifest in bulbar symptoms if involving the corticobulbar tracts or the bulbar segments of the motor cortex. A number of extrapyramidal and cerebellar conditions may also present with localization-specific (ataxic, hypokinetic, hyperkinetic) dysarthria and imaging has a role to rule out gross striatal, nigral and cerebellar pathologies. The incidental identification of tongue tumors on MRI in patients with suspected ALS has also been reported (47). A number of radiological cues have been associated with ALS, such as high signal along the pyramidal tracts on T2 weighted or FLAIR imaging, low signal in the precentral gyrus on GRE/SWI, isolated motor cortex atrophy on T1W, but these qualitative visual cues are not specific to ALS and are not sensitive for diagnostic or monitoring purposes (48). Quantitative imaging studies of ALS on the other hand have successfully captured the cortical (UMN) components of bulbar dysfunction in a somatotopic distribution (49, 50) and characterized the pathological substrate of pseudobulbar affect (51, 52). With relentless methodological (53) and conceptual advances in neuroimaging (54), the establishment of multicenter data repositories (55) and the increasing availability of 7 Tesla systems (56), the anatomical underpinnings of bulbar dysfunction are likely to be characterized in further detail.

### Auditory-Perceptual Dysarthria Evaluation and Frenchay Dysarthria Assessment

“The Mayo Clinic” method of dysarthria categorization involves auditory-perceptual evaluation of specific voice and speech features during a passage reading, phonation of /a/, and oral dysdiadochokinesis (DDK) with /pa, /ta/, /ka/, and /pataka/ (57–59). The identification of “harsh,” “strained,” or “strangled” voice quality, slow speaking rate and “excess and equal” stress pattern during passage reading and DDK are typically linked to UMN dysfunction and “spastic dysarthria.” “Breathy” or weak voice, hypernasality, nasal emissions, and articulatory imprecision without changes in speaking rate are classically associated with LMN dysfunction and “flaccid dysarthria.” ALS is typically characterized by mixed spastic-flaccid dysarthria presenting with articulatory imprecision, hypernasality, harshness, slow rate and prosodic abnormalities. Although the reliability of observational assessments have been repeatedly questioned (10), protocol standardization, assessor training, and reference samples are thought to improve assessment reliability (60). Despite these efforts, auditory-perceptual assessment remains surprisingly underutilized, requiring standardization of practices, psychometric evaluation and multi-center validation in ALS.

Tools like the Frenchay Dysarthria Assessment (FDA) (26) are particularly well-suited for diagnostic purposes as they can comprehensively assess both structure and function of the bulbar musculature through a combination of CNE items and the auditory-perceptual dysarthria assessment. However, FDA was not specifically developed for ALS, and the evaluation of its measurement properties in ALS is lacking. DDK, which is included in CNE, FDA and perceptual dysarthria assessments, is commonly used to track disease progression, and has shown high sensitivity but low specificity for detecting bulbar signs in the prodromal phase of bulbar ALS (61, 62). With further optimization, DDK may have a diagnostic potential, particularly if certain performance constrains are imposed or its complexity is increased (63, 64).

### Dysphagia Diagnosis and Screening

Videofluoroscopic swallowing evaluation (VFSE) remains the gold standard of dysphagia assessment in most neurological conditions allowing the direct visualization of swallowing safety and efficiency i.e., aspiration and the presence of residue, respectively. In ALS however, VFSE is underutilized (16) due to a number of factors such as the presumed lack of therapeutic relevance, lack of access to equipment or perceived patient burden etc. A number of screening tools have been recently evaluated for the early identification of those at risk for dysphagia in ALS. Currently, the Eating Assessment Tool-10 (EAT-10) demonstrated good sensitivity and adequate specificity for detecting aspiration in ALS (30), while instrumental measures of airflow during voluntary cough showed excellent sensitivity and specificity to detect aspiration (32). The bedside 3oz water swallow test is also extensively utilized, but its measurement properties in ALS are still unknown. There is a general consensus among SLPs that patients who fail dysphagia screening should be further evaluated by instrumental techniques to directly visualize the swallowing process using VFSE or fiberoptic evaluation of swallowing (FEES) techniques (65). This is an important consideration given the high incidence of “silent” aspiration in this patient population. Instrumental assessments, not only confirm the diagnosis of dysphagia, but inform on swallowing safety, help to identify the specific etiology of dysphagia, and guide therapeutic strategies that can be tested during the instrumental exam by directly visualizing their impact.

## Tools for Disease Monitoring—Staging and Longitudinal Tracking

Certain bulbar measures have been optimized to track the decline of bulbar function in individual patients and entire cohorts. Table 2 summarizes proposed bulbar monitoring tools in ALS.

**Table 2:** Tools to measure bulbar dysfunction severity and disease progression.

**Tool**	**Description**	**Advantages**	**Limitations**	**Recommendations**
1. ALSFRS-R, bulbar sub-score	Tracks bulbar disease progression; 3 “bulbar” questions; 0 (no function)−4 (normal function)	Quick and easy to perform; patient and caregiver versions available; well validated as a total score; (66) recent studies suggest using “domain-specific” subscores instead of a total score; (67, 68) declines linearly; an accepted end point in clinical trials	Limited assessment of bulbar dysfunction; symptom report; may underestimate sisease severity; (69) changes relatively late in the disease; skewed to detection of the LMN impairment (70)	Recommended for use in clinic and clinical trials but caution due to limited nature of bulbar assessment
2. Center for Neurologic Study-Bulbar Function Scale (CNS-BFS)	Reports solely on bulbar symptoms, 21 questions regarding speech, swallowing and salivation	Validated-high criterion and construct validity, (71) good reliability, responsive to change over time and improved in a clinical trial (72)	Symptom report; potentially not sensitive to early phases of the disease; need further validation against VFSE	Recommended for bulbar evaluation in clinic and clinical trials
3. Appel scale	Includes 5-point ratings of functional status of speech and swallowing (scores 6–30) and bulbar disease progression	Reliable; responsive to disease progression (linear decline); the composite score distinguishes slow from fast progressors, predicts survival, provides bases for clinical classification with management recommendations depending on severity (73)	Validation is limited to date; includes only 2 questions related to bulbar function-−1 speech and 1 swallowing	Requires further evaluation of measurement properties; limited for the assessment of bulbar dysfunction
4. Norris scale	34-item ranking system; (74) 6 items in the “bulbar” category (i.e., chew, swallow, speak, jaw jerk, atrophy face/ tongue, lability) on a 3-point scale for each item	Quick and easy to administer; includes a range of bulbar items; has been used in clinical trials (75)	All items (functional and non-functional) rated equally; 3-point scale might be too coarse to detect change; limited information on the development and validation of the tool; responsiveness not established	Requires further evaluation of measurement properties; limited for the assessment of bulbar dysfunction
5. ALS Severity Scale (ALSSS)	10-point staging scale; was designed to supports management/rehabilitation practices in ALS; includes 1 speech and 1 swallowing item (0 to 10)	Easy to perform; clear description of each stage; adequate reliability; sig correlations with timed tests and speech intelligibility; responsive to change over time (76)	Ordinal scale; includes only 1 speech and 1 swallowing item	Requires further evaluation of measurement properties
6. Neuromuscular Disease Clinical Status Scale (NdSSS)	8-stage dysphagia severity scale to track the development of symptoms of dysphagia over time	Quick and easy to administer by a trained clinician; reliability, concurrent validity relative to other scales and responsiveness reported (77) and adequate	Focused predominantly on description of intake/ diet; not validated against VFSE	Although promising, requires further evaluation of measurement properties in other cultures
7. Oral Secretion Scale (OSS)	5-point scale to evaluate the severity of sialorrhea in ALS	Quick and easy to administer; validated against ALSFRS-R bulbar subscore and SSS; adequate reliability; can be used by different professions (78)	Floor effect in the more severely involved individuals; responsiveness not assessed; not linked to dysphagia outcomes	Recommended for evaluation of severity of sialorrhea in clinic and in clinical trials
8. Sialorrhea Scoring Scale (SSS)	9-point scale to evaluate the severity of sialorrhea	Quick and easy to administer; validated against ALSFRS-R bulbar subscore and OSS; adequate reliability; can be used by different professions; better spread of scores across the severity range compared to OSS (78)	Responsiveness not assessed; reliability was somewhat lower than for OSS; not linked to dysphagia outcomes	Recommended for evaluation of severity of sialorrhea in clinic and in clinical trials
9. Sentence Intelligibility Test—Speech Intelligibility and Speaking rate	% of words understood by a listener during a sentence transcription task, and number of words produced per minute (WPM)	Easy to perform; supported by software; validated in multiple studies with respect to ALSSS (79) and ALSFRS-R; decline in rate to 125 WPM predicts intelligibility drop and is used to time AAC interventions; WPM changes linearly with disease progression (80, 81)	Requires a trained SLP; requires a trained transcriber; low sensitivity to early bulbar disease; (61) declines over 12% in sentence intelligibility and 37 WPM are outside of measurement error (82)	Speaking rate is recommended to be tracked during clinic in order to plan AAC interventions for those at risk for loss of speech intelligibility
10. Timed tests: Speech and pause durations in a passage^*^	A passage reading task (e.g., Bamboo) (83) allows a separation between speaking and pause events; gives a detailed picture of the components of speaking rate	Easy to perform; allows practice to minimize reading errors; distinguished patients with ALS with bulbar and respiratory signs; (84) showed sensitivity to change in a drug trial (85)	Currently requires time consuming, by-hand measurements; requires training; measurement properties (e.g., responsiveness, measurement error) are not well established; bulbar effects need further differentiation from respiratory and cognitive effects	Requires further standardization as well as better testing of measurement properties; subsequently would benefit from automation
11. Times tests: DDK^*^	A syllable repetition task (pa; ta; ka; pa-ta-ka) in syllables per second (syl/sec) that is used to detect slowing of the oral movements	Easy to perform; clinicians are familiar with the task; easily measured instrumentally; free of cognitive-linguistic effects; distinguishes slow from fast progressors; (86) cut off 4.6 syl/s 91% sensitivity and 54% specificity in detecting bulbar signs in pre-symptomatic patients (61)	Requires training/ modeling and maximum effort from patients; measurement properties are not fully established (e.g., responsiveness; error of measurement)	Requires further standardization as well as better testing of measurement properties; subsequently would benefit from automation
12. Maximum Tongue Pressure (MTP)^*^	A measure of tongue strength using a commercially available devices	Affordable easy to use clinical tool; validated against ALSFRS-R bulbar subscore and VFSE; cut off < 21 KPa has sensitivity 80% and specificity 100% for detecting bulbar dysfunction on ALSFRS-R (87) and oral dysphagia; adequate reliability; independent prognostic factor of survival (88)	Requires training of the clinician and patient prior to measurement—results are placement dependent; (89) requires max effort; insufficient data on responsiveness; (90) not associated with dysarthria and speech intelligibility loss	Requires further standardization as well as testing of measurement properties

* *May be used for diagnostic purposes*.

### Bulbar Monitoring (Overall)

A recent clinical practice survey of ALS care in the United States revealed that the Revised ALS—Functional Rating Scale (ALSFRS-R) bulbar sub-score, clinician or patient administered, represented the only measure routinely used to evaluate bulbar dysfunction in the clinical setting (16). It contains only 3 questions to address changes in speech, swallowing and salivation that are each merely rated on a four-point ordinal scale. While the total ALSFRS-R score is thought to have excellent reliability, the measurement properties of the individual sub-scores (e.g., bulbar) have not been specifically evaluated to date (66, 67, 91, 92). The Center for Neurologic Study-Bulbar Function Scale (CNS-BFS) is a 15-item questionnaire of bulbar involvement which has recently been validated against the ALSFRS-R and “timed” speech and swallowing tasks, and has already been successfully utilized in a clinical trial (71, 72). However, the CNS-BFS still needs to be validated against VFSE.

The Appel scale is one of the best characterized tools to track ALS-associated impairment and functional decline (73). Other clinician-administered instruments include the Norris (74), Tuffs (93), and Charing Cross (94) scales, but their original development, optimization and validation studies can be difficult to acquire and subsequently, their performance is relatively difficult to judge. The ability of these instruments to represent specific stages and their potential to track progressive bulbar impairment is largely unknown. A number of global ALS staging systems have been developed recently, such as the King's clinical staging system, the Milano-Torino (MiToS) functional staging, the Fine'til 9 (FT9) framework (95, 96), but bulbar impairment is just a small component of these instruments. Among the staging tools, the ALS Severity Scale (ALSSS) is particularly noteworthy, as it uses a 10-point scale for two bulbar functions, speech and swallowing. It was designed to guide rehabilitation efforts in ALS and, and pending formal psychometric evaluation, it may prove to be particularly useful (97).

### Functional Monitoring of Dysphagia and Oral Secretion Scales

The Neuromuscular Disease Clinical Status Scale (NdSSS), which focuses solely on dysphagia, underwent one of the most rigorous psychometric evaluations to date. This tool exhibited excellent inter- and intra- rater reliability and correlated well with the functional oral intake scale (77). It has not been validated against VFSE yet, and given the potential for considerable geographical differences in oral intake, it is unclear how this tool may be validated around the world. While there are several tools to assess sialorrhea in ALS, such as the Oral Secretion Scale (OSS) and Sialorrhea Scoring Scale (SSS) available (78), these also need comprehensive psychometric evaluation and validation.

### Functional Monitoring of Dysarthria

“Speech intelligibility” refers to the degree to which a speaker is understood by a listener, and “speaking rate” refers to speaking speed. Although both of these measures can be assessed on a 5 or 7-point Likert scale (49), the Sentence Intelligibility Test (SIT) is often preferred by SLPs, as it provides a more fine-grained estimate of speech intelligibility (i.e., percent of words transcribed correctly) and speaking rate (i.e., number of words produced per minute) (98). Speech intelligibility is considered abnormal when it falls below 97%, and speaking rate is considered abnormal below 160 words-per-minute (WPM) (99, 100). Speech intelligibility is a general indicator of the severity of dysarthria and it declines relatively late in the course of the disease (101). Speaking rate typically declines prior to significant changes in speech intelligibility, and it changes more linearly with symptom duration than speech intelligibility. Therefore, speaking rate is particularly useful in monitoring bulbar impairment longitudinally (102, 103). A speaking rate of 125 WPM or less is the recommended cut off for to trigger referral to the augmentative and alternative communication services (99).

Digital speech recordings and automated analyses can provide new opportunities for in-depth, observer-independent evaluations, especially during a passage reading and syllable repetition (DDK) tasks. In passage reading tasks, such as the Bamboo Passage, which has been specifically developed to support automatic analyses, certain phrases are semi-automatically identified, and speech duration and pause intervals can be accurately quantified (83). The measures derived from this analysis e.g., percentage pause time, mean phrase duration etc. have been identified to be sensitive to the prodromal stages of bulbar dysfunction (61) and also showed to detect response to pharmaceutical interventions such as dextromethorphan/quinidine (Nuedexta) therapy (85). A recent longitudinal study suggested that the main advantage of the DDK tasks may be in their ability to reliably distinguish slow- and fast-progressors (86).

### Physiological Monitoring

Muscle strength testing in ALS has been initially performed using force transducers (strain gauges) (104, 105) and later with pressure bulbs via the Iowa Oral Performance Instrument (IOPI) (IOPI Medical LLC) or TPM-01 (JMS, Hiroshima). Lingual pressure testing using the IOPI revealed adequate reliability of a maximum tongue pressure estimate (MTP, or maximum anterior isometric pressure, MAIP) but not for the measure of endurance (89). Only one study assessed longitudinal changes in MTP in ALS to date (90) and reported its decline in patients with bulbar onset within 3 months and for those with spinal onset within 6 months. Tongue strength has also been shown to be an independent predictor of survival (88); however, formal psychometric evaluation is awaited to determine the MTP's utility to measure progressive changes over time.

## Discussion

In order to firmly establish the clinical utility of specific bulbar instruments and their potential as outcome measures in clinical trials, their measurement properties need to be comprehensively characterized. Among the diagnostic dysphagia instruments, screening tools, such as EAT-10 and voluntary cough (30, 32) have been well evaluated. Among speech measures, only DDK rate came close to demonstrating diagnostic utility (61). The remaining tools require extensive evaluation with regards to their diagnostic accuracy. While a large number of novel assessment tools have been proposed to track the progression of bulbar impairment, only the ALSFRS-R, the CNS-BFS and some bulbar staging systems (e.g., NdSSS, OSS, SSS) meet at least basic measurement requirements. Most existing disease monitoring tools lack the ability to capture subtle progressive changes, which is indispensable for disease tracking tools. Robust systematic psychometric evaluation is needed to improve the currently available clinical, academic and pharmacological-trial assessment tools.

Despite the gaps in the current literature and the limitations of current clinical trial designs, we are likely to witness considerable advances in standardized bulbar assessments and the emergence of purpose-designed, disease-specific, well-validated bulbar assessment tools. Emerging technologies such as quantitative neuroimaging, muscle ultrasound, electrical impedance myography (EIM), high-resolution manometry, videomanofluoroscopy, and speech acoustic monitoring are likely to soon complement our armamentarium of clinical tools. A number of promising imaging techniques have already been utilized to characterize the pathological substrate of bulbar impairment in ALS including diffusion tensor imaging (106, 107), cortical thickness measurements (50, 108, 109), morphometry-type analyses (49, 110), magnetization transfer ratio imaging (106), MR spectroscopy (111), MRI intensitometry (112), and task-based functional MRI (113, 114). Despite these advances, MRI-derived metrics remain underutilized in the clinical setting and as outcome measures in pharmacological trials. This is in sharp contrast with clinical trials in Multiple Sclerosis, where MRI plays an established role as a key outcome measure in phase III clinical trials (115). Muscle ultrasound may capture tongue fasciculations in the absence of fasciculation potentials on EMG and the combination of ultrasound and EMG may help the detection of early denervation (116). Likewise, EIM shows promise in detecting changes in the structural composition of the tongue in ALS and may evolve into an important tool to detect early bulbar involvement (117, 118). High-resolution manometry and videomanofluoroscopy may provide unique insights into the dynamics of bolus movement and swallowing pressures enabling early detection of bulbar dysfunction and thus, timely interventions (119, 120). Acoustic analysis of speech has been proposed as a means for the objective assessment of bulbar impairment for over two decades, but until recently extracting these measure has been extremely time consuming. Recent developments in automatic audio and video analysis methods and smart phone technologies make speech analysis technologically feasible, enabling observer-independent multiparametric analyses (121–123). These emerging methodologies will need careful development, optimization and evaluation according to established methodological guidelines (e.g., COSMIN framework).

## Conclusions

Recent advances in neuroimaging, development of staging systems, patient-reported outcome measures and the emergence of novel instrumental speech and swallowing assessment techniques promise novel insights into bulbar dysfunction in ALS. However, in order for these methods to be integrated into routine clinical practice and pharmacological trials, they have to be rigorously evaluated with respect to their measurement properties, diagnostic performance and longitudinal tracking abilities. The establishment of large international collaborations and relentless biomarker research efforts give cause for optimism for the development of validated bulbar assessments, which in turn will contribute to best practice recommendations, enable well-timed clinical interventions and facilitate accurate patient stratification in clinical trials.

## Author Contributions

YY and PB reviewed references, drafted the manuscript, and generated the tables. EP provided expert opinion on dysphagia assessments and edited the drafts of the manuscript. JG provided expert opinion on dysarthria assessments and edited the drafts of the manuscript. CB provided expert opinion on measurement development and edited the drafts of the manuscript.

### Conflict of Interest Statement

The authors declare that the research was conducted in the absence of any commercial or financial relationships that could be construed as a potential conflict of interest.

## Abbreviations

AAC: augmentative alternative communication
ALS: Amyotrophic Lateral Sclerosis
ALSFRS-R: Amyotrophic Lateral Sclerosis—Functional Rating Scale—Revised
ALSSS: ALS Severity Scale
CNS-BFS: Center for Neurologic Study-Bulbar Function Scale
COSMIN: COnsensus-based Standards for the selection of health Measurement Instruments
CNE: Cranial nerve exam
DDK: dysdiadochokinesis
EAT-10: Eating Assessment Tool-10
EIM: electrical impedance myography
EMG: electromyography
FLAIR: Fluid-attenuated inversion recovery
FDA: Frenchay Dysarthria Assessment
FEES: fiberoptic evaluation of swallowing
FSE: Videofluoroscopic swallowing evaluation
FT9: Fine'til 9
GRE/SWI: gradient recalled echo/susceptibility weighted imaging
IOPI: Iowa Oral Performance Instrument
KPa: kilopascal
LMN: lower motor neuron
MAIP: maximum anterior isometric pressure
MiToS: Milano-Torino staging
MR: magnetic resonance
MRI: magnetic resonance imaging
MUNE: Motor Unit Number Estimation
MTP: maximum tongue pressure
MUNIX: Motor unit number index
MUPs: motor unit potentials
NdSSS: Neuromuscular Disease Clinical Status Scale
OSS: Oral Secretion Scale
SCM: sternocleidomastoid
SLPs: speech-language pathologists
SSS: Sialorrhea Scoring Scale
SIT: Sentence Intelligibility Test
syl/sec: syllables per second
T1 W: T1 weighted
UMN: upper motor neuron
WPM: words-per-minute.
